# Determinants of Longitudinal Changes in Exercise Blood Pressure in a Population of Young Athletes: The Role of BMI

**DOI:** 10.3390/jcdd12020074

**Published:** 2025-02-15

**Authors:** Francesca Battista, Marco Vecchiato, Kiril Chernis, Sara Faggian, Federica Duregon, Nicola Borasio, Sara Ortolan, Giacomo Pucci, Andrea Ermolao, Daniel Neunhaeuserer

**Affiliations:** 1 Sports and Exercise Medicine Division, Department of Medicine, University of Padova, Via Giustiniani 2, 35128 Padova, Italy; francesca.battista@unipd.it (F.B.); kiril.chernis@studenti.unipd.it (K.C.); sara.faggian.3@phd.unipd.it (S.F.); federica.duregon@unipd.it (F.D.); nicola.borasio@phd.unipd.it (N.B.); sara.ortolan@aopd.veneto.it (S.O.); andrea.ermolao@unipd.it (A.E.); daniel.neunhaeuserer@unipd.it (D.N.); 2 Clinical Network of Sports and Exercise Medicine of the Veneto Region, 35131 Padova, Italy; 3 Institute of Mountain Emergency Medicine, EURAC Research, Viale Druso 1, 39100 Bolzano, Italy; 4 Unit of Internal and Translational Medicine, Terni University Hospital, 05100 Terni, Italy; giacomo.pucci@unipg.it; 5 Department of Medicine, University of Perugia, 06123 Perugia, Italy

**Keywords:** cardiovascular risk, obesity, hypertension, exercise test

## Abstract

AIM: Higher exercise blood pressure in adults correlates with many cardiometabolic markers. The aim of this study was to investigate the main determinants of longitudinal variations in exercise blood pressure in young athletes. METHODS: A longitudinal retrospective study was conducted on adolescent athletes who underwent at least two sport-related pre-participation screening visits, including exercise testing with a standardized incremental ramp protocol on treadmill. Blood pressure was assessed at rest (SBP_rest_), at the 3rd minute of exercise (SBP_3min_), and at peak exercise (SBP_peak_). Predictors of blood pressure response (i.e., respective changes vs. baseline (Δ)) were determined by multivariate regression models after adjustment for age, sex, follow-up duration, related baseline SBP values, characteristics of sport, and ΔBMI. RESULTS: A total of 351 young athletes (mean age at baseline 13 ± 2 years, 54% boys, average follow-up duration 3.4 ± 2.2 years) were enrolled. BMI increased by 1.5 ± 1.8 kg/m^2^ (*p* < 0.001) during follow-up. At baseline, mean SBP_rest_ was 103 ± 14 mmHg, mean SBP_3min_ 124 ± 18 mmHg, and mean SBPpeak 154 ± 23 mmHg. A significant between-visit increase in SBP_rest_ (ΔSBP_rest_ 7.0 ± 17.4 mmHg; *p* < 0.001), ΔSBP_3min_ (4.8 ± 11 mmHg, *p* < 0.001), and ΔSBP_peak_ (11.7 ± 24 mmHg, *p* < 0.001) was observed. ΔSBP_3min_ was significantly predicted by male sex (*p* < 0.01), baseline BMI (*p* < 0.01), ΔBMI (*p* < 0.01), and number of practiced sports (*p* < 0.05), whereas ΔSBP_peak_ was positively predicted by male gender (*p* < 0.01), baseline BMI (*p* < 0.05), and ΔBMI (*p* < 0.01) and negatively by baseline resting heart rate (*p* < 0.01). In a logistic regression model, ΔBMI was the only independent determinant of passing from a lower to an upper quartile of SBP_3min_ (*p* < 0.001), while ΔBMI and male sex were independent determinants of moving to a higher quartile of SBP_peak_ (*p* < 0.001). CONCLUSIONS: Increase in BMI during development and male sex are independent determinants of the increase in exercise blood pressure, both at light and maximal intensity, in a population of adolescent athletes.

## 1. Introduction

Elevated blood pressure (BP) is nowadays a leading risk factor for cardiovascular disease worldwide [1], affecting about 36% of females and 41% of males in European Society of Cardiology (ESC) member countries [2]. A hypertensive response to exercise has been shown to predict the future onset of overt hypertension, irrespective of apparently normal BP at rest [3,4,5]. Moreover, systolic blood pressure (SBP) at submaximal intensity assessed during early stages of an incremental exercise stress test significantly predicts the presence of hypertension, confirmed by 24 h ambulatory blood pressure monitoring in adults [6]. Exaggerated exercise blood pressure recorded both at light and at maximal intensity of exercise is associated with an increased risk of cardiovascular events and mortality in adults, independently from resting blood pressure and other conventional risk factors [7,8]; this has also been linked to arterial stiffness and cardiorespiratory fitness [9]. Therefore, an abnormal increase in systolic blood pressure during exercise may act as a potential forewarning to clinicians of an increased cardiovascular risk, irrespective of resting blood pressure [10]. Independently, whether defined on the basis of an exaggerated change in systolic blood pressure from rest, as absolute values during exercise testing, or by evaluating the recovery phase after moderate intensity exercise, exaggerated exercise blood pressure predicts future incidence of hypertension independently of baseline resting BP [11]. Considering that maximal exercise intensity is seldomly reached in everyday life by the general population, measurement of blood pressure during light to moderate intensity may be a more suitable tool to assess the true risk related to elevated BP and also because this value would be comparable to the chronic BP load during daily life activities [12]. It has been shown that SBP recorded at light-intensity exercise is an independent predictor of masked hypertension and also correlates significantly with 24 h systolic blood pressure monitoring, which is the strongest blood pressure measure in predicting target organ damage and cardiovascular outcomes in hypertension [12]. Consequently, exaggerated exercise blood pressure might itself be associated with hypertension-related organ damage [13,14] in pre-hypertensive [15] and hypertensive patients [16]. Furthermore, it must be noted that an exaggerated exercise blood pressure response is a common finding also in healthy normotensive populations [17], although it is more prevalent in people with elevated cardiovascular risk (i.e., up to 50% of patients with type 2 diabetes mellitus) [18]. In normotensive subjects, systolic blood pressure recorded during light-intensity exercise has been correlated with left ventricular mass as a marker of target organ damage, independently from age, systolic blood pressure at rest, and body mass index (BMI) [19]. On the other hand, in a large cohort of adults from the Framingham Heart Study, it was found that higher exercise systolic blood pressure was related to older age, female sex, higher baseline resting systolic blood pressure and heart rate, new-onset of type 2 diabetes mellitus, and also BMI. Particularly, an increase of 1 kg/m^2^ in BMI during follow-up examinations was associated with a 1 mmHg higher exercise systolic blood pressure [20]. Currently, about 54.8% of people in ESC member countries live with overweight and 17.0% with obesity, without significant differences between middle- and high-income countries, leading to an associated risk of death of more than 13% across the European region [2]. Obesity is a chronic and relapsing disease that is often complicated by hypertension, dyslipidemia, insulin resistance, endothelial dysfunction, and inflammation which together increase the risk of cardiovascular diseases and death [21]. Recent reports indicate that about 8% of children younger than 5 years of age have obesity [2] and that this obesity pandemic has significantly increased in young people after the COVID-19 pandemic [22,23,24]. Obesity is particularly concerning during developmental age because of metabolic changes resulting in a significant increase in cardiovascular risk in adulthood and alterations in body composition and bone metabolism that may also be accentuated by therapeutic strategies for obesity such as bariatric surgery if not specifically addressed with physical exercise, nutrition, and specific contrasting measures [25]. Indeed, new ESC guidelines for the management of elevated blood pressure and hypertension markedly emphasize that the relationship between blood pressure values and global cardiovascular risk is continuous and starts long before reaching the threshold values for the diagnosis of hypertension [26]. For all these reasons, it is crucial to focus the medical efforts on early prevention and detection of preclinical onset of risk factors and early markers of cardiometabolic disease, especially in childhood and adolescence, where prevention should be a health priority and when interventions are easier and more effective.

Little information is currently available about the possible association between exercise blood pressure in adolescents and the cardiovascular risk profile in adulthood. Our aim is, therefore, to explore determinants of exercise blood pressure values both at light-intensity exercise and at maximal exercise in physically very active adolescents.

## 2. Materials and Methods

### 2.1. Population and Assessments

This is a retrospective study consecutively enrolling adolescent subjects presenting from 2014 to 2017 at the Sport and Exercise Medicine Division of the Padova University Hospital for a mandatory clinical evaluation, part of the pre-participation screening of athletes, which in Italy requires an annual exercise stress testing. Subjects were included after they had been evaluated at least twice, considering the two more spread-out recorded visits. Exercise stress testing was carried out by expert medical staff. Patients underwent a treadmill exercise test (treadmill Marquette T-2000 Series, with Case 8000 stress system, General Electrics, Waukesha, WI, USA) by using an adapted and standardized incremental exercise protocol modified from the Bruce protocol [27,28]. Subjects that performed submaximal tests, used anti-hypertensive treatment, or had symptoms during testing were excluded. Moreover, this protocol foresees a gradual and progressive increase in the slope and speed, with continuous 12-lead electrocardiographic monitoring. The stress test was considered valid if the patient reached a rate of perceived exertion (RPE) of at least 18/20 according to the Borg Scale, associated with a heart rate higher than 85% of the maximum predicted heart rate calculated by age (Fox’s formula 220—age) [29]. The duration of the incremental exercise test depended on the subject’s characteristics and functional capacity. The stress test included at least four minutes of a monitored recovery phase. The blood pressure at rest was measured by expert medical or nursing staff before the stress test in a quiet room, in seated position, after at least five minutes of rest by using a mercury sphygmomanometer with an auscultatory method. The size of the cuff used for pressure measurement was chosen based on the patient’s arm diameter. Blood pressure values were recorded also during the stress test, in particular at submaximal intensity at the 3rd minute of exercise (estimated exercise intensity equal to six METs, using the American College of Sports Medicine’s formula corrected following Foster’s suggestions) [30], at the maximal effort, and in supine condition immediately after stopping the exercise phase and every minute during recovery period. During the stress test, blood pressure was measured by auscultatory method considering the first Korotkoff tone for the systolic value and the attenuation phase for the diastolic value. The diagnosis of normal, high-normal blood pressure, and hypertension condition was performed by using the 2016 European Society of Hypertension guidelines for the management of high blood pressure in children and adolescents, reporting arterial blood pressure values into reference percentiles for subjects under 16 years of age [31]. The calculation of the respective percentile of blood pressure was based on the 2017 American Academy of Pediatrics guidelines reference population, which did not consider adolescents with obesity, thus fitting better with a population of healthy adolescents [32]. Anthropometric data were collected by expert medical or nursing staff before the stress test according to standard procedures [33]. Body mass index (BMI) was calculated as the ratio between weight (expressed in kilograms) and squared height (expressed in centimeters). Overweight and obesity were diagnosed on the basis of the World Health Organization definition for adults and adolescents [34,35].

### 2.2. Statistical Analyses

Descriptive statistics are expressed as means and standard deviations for continuous variables and as counts and percentages for categorical variables. We used *t*-tests to determine the significance of differences among demographic and continuous variables. We determined the receiver operating characteristic (ROC) curve to evaluate the predictive power of systolic blood pressure at rest (SBP_rest_), systolic blood pressure at 3rd minute of exercise (SBP_3min_), and systolic blood pressure at peak exercise (SBP_peak_) for the diagnosis of hypertension at follow-up. To create the ROC curve, subjects with overt diagnosis of hypertension were excluded. We calculated changes in risk factors for continuous variables. Primary outcomes were the main determinants of the difference between visit 1 (V1) and visit 2 (V2) in SBP_3min_ (ΔSBP_3min_) and SBP_peak_ (ΔSBP_peak_). We used partial correlation analyses to investigate the correlation between clinical characteristics and ΔSBP_3min_ and ΔSBP_peak_, corrected for baseline values and possible confounders. We calculated quartiles of SBP_3min_ and SBP_peak_ at visit 2; subsequently, we used multiple linear regression to find determinants of the likelihood of being in the higher quartile of SBP_3min_ and SBP_peak_ at V2. A two-tailed *p* < 0.05 was considered statistically significant. All analyses were performed with the use of SPSS software (SPSS version 23, SPSS, Inc., Chicago, IL, USA). Researchers were not blinded during study analyses.

## 3. Results

The main characteristics of the population at baseline (visit 1) and at follow-up (visit 2) are summarized in Table 1. The population was composed by 351 very active (mean weekly training time 345.2 min) adolescents (mean age 13.0 ± 2 years), with 54% of them males. The mean follow-up was up to 3.4 years (from 1 to 14 years).

**Figure 1:**
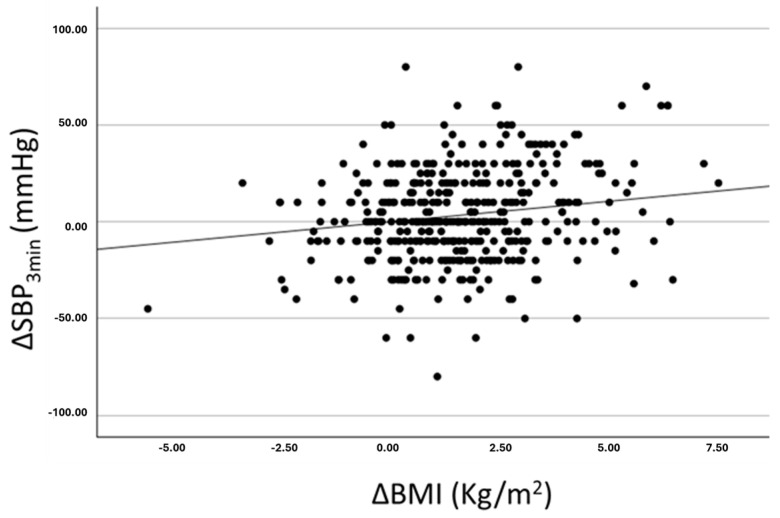
Correlation between change over time of systolic blood pressure recorded at light-intensity exercise (ΔSBP_3min_) and the change over time of BMI (ΔBMI).

At rest at visit 1, the mean of systolic and diastolic blood pressure (SBP_rest_) values were, respectively, 103 and 55 mmHg (±14/11 mmHg). Additionally, 4.8% of subjects showed high-normal blood pressure values, while 4.8% were in the hypertensive range at visit 1. The change in SBP_rest_ during follow-up was equal to +7 (±17.4) mmHg, with the difference between the means at visit 1 and visit 2 proving statistically significant (*p* < 0.001). We can also highlight a significant increase in systolic blood pressure (SBP) at light-intensity exercise (SBP_3min_) corresponding to +4.8 (±11) mmHg and at peak-intensity exercise (SBP_peak_) corresponding to +11.7 (±24) mmHg. The mean value of BMI at visit 1 was 20.2 (±3.1), with a mean BMI percentile equal to 54.1 (±26.3), while 3% of the population fell within the range of obesity, and 6% was overweight. The change in BMI during follow-up was equal to +1.5 (±1.8) kg/m^2^, resulting in a statistically significant difference (*p* < 0.001) and corresponding to the −1.0 percentile without reaching statistical significance. The receiver operating characteristic (ROC) curve showed that SBP_3min_ (*p* < 0.02) and SBP_peak_ (*p* < 0.01) significantly predicted diagnosis of hypertension at follow-up. SBP_rest_ did not show any significant predictive power on incident diagnosis of hypertension (*p* = 0.10). In a partial correlation analysis (Table 2) that corrected for basal values of SBP_3min_, age at visit 1, and duration of follow-up, the change in SBP_3min_ (ΔSBP_3min_) was significantly higher in males (R 0.30; *p* < 0.01), in subjects with higher BMI (R 0.24; *p* < 0.01), in subjects who practiced a greater number of sports (R 0.11; *p* < 0.05), and in those with a greater increase in BMI (R 0.18; *p* < 0.01). The relationship between ΔSBP_3min_ and ΔBMI is also reported in Figure 1.

The partial correlation analysis, which refers to the change in SBP_peak_ (ΔSBP_peak_) after correction for basal values of SBP_peak_, age at visit 1, and duration of follow-up (Table 3), was direct and significant for male sex (R 0.38; *p* < 0.01), BMI (R 0.13; *p* < 0.05), and its increasing during follow-up (R 0.21; *p* < 0.01) as well as indirect and significant for resting heart rate (HR) at visit 1 (R −0.19; *p* < 0.01). The changes of SBP_rest_, SBP_3min_, and SBP_peak_ did not differ between hypertensive and normotensive subjects.

In a logistic regression model (Table 4), the likelihood of being included in a quartile of SBP_3min_ higher than those at baseline was independently determined by the increase in BMI (*p* < 0.001). In a linear regression model the likelihood of being included in a higher quartile of SBP_peak_ was independently determined by the male sex and the increase in BMI (*p* < 0.001).

## 4. Discussion

The main result of this study is that, in a population of very active adolescents, the change over time of systolic blood pressure recorded at light-exercise intensity (ΔSBP_3min_) is significantly determined by the change over time of BMI (ΔBMI).

This relationship seems to be independent from many covariates, such as age, duration of follow-up, and BMI at visit 1. In addition, it should be noted that the average BMI and also BMI percentiles were in line with normal weight both at baseline and follow-up, showing a low prevalence of obesity in this population. Therefore, this correlation refers to BMI as a continuous variable and emphasizes that weight gain may be associated with an increase in blood pressure even at a preclinical stage, i.e., before obesity occurs. This finding is similar to other previous studies conducted in adult populations, i.e., that from Framingham offspring, in which was found that the greater the weight gain in time, the higher the follow-up exercise systolic blood pressure [20]. Similarly, in the linear regression analysis, ΔSBP_peak_ was also shown to be determined by the change in BMI over time. In observational studies in adults, the relationship between BMI and exercise blood pressure seems to be linked to an impaired cerebral blood flow velocity [36]. In children with type 2 diabetes mellitus, regardless of glucose tolerance status, subjects with overweight and obesity displayed higher absolute systolic and diastolic blood pressure for a given workload despite a similar heart rate. Based on these results, data suggest that the blood pressure response to exercise among adolescents is more dependent on body weight than on glucose tolerance [37]. In the Quebec Family Study, in a cohort of Canadian adolescents, multiple linear regression analysis revealed that BMI was a primary determinant of systolic blood pressure (β = 0.9–1.1, *p* < 0.001). On the basis of this and other studies, in a review of the literature, Torrance et al. emphasized that overweight may be a key determinant of elevated systolic blood pressure in children [38]. Indeed, particularly in children and adolescents, body weight was shown to be the critical predictor of cardiovascular health and was also a stronger predictor than cardio-respiratory fitness, challenging the slogan “fat but fit” and suggesting that despite high cardiorespiratory fitness due to young age and active lifestyle, BMI remains a key component for cardiometabolic health in adolescents [39]. Nevertheless, to the best of our knowledge, there are currently no longitudinal studies examining the association between the blood pressure response to exercise in children or adolescents and cardiovascular risk in adulthood. The mechanisms of an abnormal elevation of exercise BP remain unclear but are likely to be multifactorial [12]. There are several possible explanations, including the hypothesis that a higher body weight may promote a stiffer arterial system. Furthermore, Park et al. showed that insulin resistance was significantly raised in those with an exaggerated BP response to exercise, and respective metabolic markers predicted the presence of a hypertensive response to exercise independently of age, sex, body mass, and baseline systolic BP [40]. In our study, ΔSBP_peak_ was independently determined also by male sex. It is widely known that cardiovascular risk factors and their distribution are sex-specific, and particularly among young people, male sex is a key component for the development of hypertension [26]. Even more worthy of attention is the higher prevalence of males among metabolically unhealthy people and that, in young adults, the progression from a metabolically healthy to unhealthy profile is more common in men than women [41]. The combination of metabolic alterations such as dyslipidemia and insulin resistance could impair vascular reactivity, increase vascular resistance during exercise, and contribute to an abnormal increase in BP with exercise [42,43]. Failure of the cardiovascular system in counteracting excessive rises in arterial BP and decreasing left ventricular afterload during exercise may elevate input impedance (ratio of pulsatile pressure to pulsatile flow) and cause a greater rise in systolic BP [44]. On the other hand, impaired endothelium-dependent vasodilatory function could be a further underlying contributory mechanism [45]. Children with overweight have an increased resting cardiac output that is mainly due to expanded stroke volume [46,47]. Another possible involved mechanism is an increased sodium sensitivity that could cause a plasma volume-dependent increase in stroke volume and thus cardiac output, thereby increasing BP [46]. Several studies have suggested that an activation of the sympathetic nervous system appears to be a leading candidate to explain the relationship between obesity and hypertension in humans [48].

By excluding overt diagnosis of hypertension at visit 1, the office systolic blood pressure at rest did not predict incident diagnosis of hypertension during follow-up, and consistently, in the partial correlation analysis, we found that the resting BP at baseline was not significantly correlated with the changes over time in systolic blood pressure during exercise both at low intensity (ΔSBP_3min_) or at peak exercise (ΔSBP_peak_). On the other hand, measures of exercise blood pressure were significantly predictive of diagnosis of hypertension at visit 2. Previous observational studies have found that an exaggerated response to light-intensity exercise (expressed as variations of systolic blood pressure from rest to the exercise phase) seemed to be associated with left ventricular hypertrophy in normotensive middle-aged women [49]. Schultz et al. found that the systolic BP response at early stages of an exercise stress test was more strongly associated with the presence of hypertension as confirmed by 24 h ambulatory BP measurement when compared with maximal exercise blood pressure. The exaggerated exercise blood pressure response, particularly at light-intensity exercise, may have an important relevance to both clinical and athletes’ exercise stress testing. In clinical practice, this finding may reveal hypertension-related cardiovascular risk factors that would remain otherwise undetected by standard screening measures [6]. Currently, the main limit to the use of this parameter in clinical practice is the lack of universally recognized normality values for exercise blood pressure in adults and adolescents as well as the scarcity of longitudinal studies that define the actual attributable risk of any health/disease-related outcome to this parameter.

### Limitations and Perspectives

The main limitation of this research work is the possible confounding power of the pressure recording modality, which may lead to a slight “digit preference” bias. Furthermore, cardiovascular risk factors have not been recorded in a standardized way because of the clinical nature of this study. Finally, long-term data on the development of hypertension and other cardiovascular risk factors in adulthood would allow stronger conclusions leading to clinical implications in primary and secondary prevention. Furthermore, the application of advanced artificial intelligence tools may, in the future, offer new insights into the complex mechanisms underlying variations in exercise blood pressure, enabling more precise and personalized predictive analyses [50].

## 5. Conclusions

This paper shows that the increase in BMI over time and the male sex are independent determinants of the increase in exercise blood pressure recorded both at submaximal and maximal exercise intensity in a population of young and apparently healthy athletes. These findings suggest that attention should be paid to BMI in young people to prevent the rise in overweight and obesity, with awareness of the continuous relationship among BMI, blood pressure, and the development of cardiometabolic diseases. The contribution of this study is that it adds original information on the possible determinants of elevated exercise blood pressure and proposes submaximal exercise blood pressure evaluation as a potential screening tool and early marker of cardiovascular risk in an understudied population such as adolescents. It needs to be further investigated whether this is associated with a higher risk of arterial hypertension in adulthood.

## Figures and Tables

**Table 1:** Main characteristics of the population at baseline (visit 1) and at follow-up (visit 2).

	Visit 1	Visit 2	Mean Change	*p*
Age (years)	13 (±2)	16.9 (±3)	+3.5 (±2.2)	
Height (cm)	162.5 (±12.1)	170.4 (±10.3)	+7.9 (±9.2)	<0.0001
Weight (Kg)	54.2 (±13.9)	63.8 (±12.7)	+9.5 (±9.1)	<0.0001
BMI (Kg/m^2^)	20.2 (±3.1)	21.7 (±3.1)	+1.5 (±1.8)	<0.0001
BMI percentiles *	54.1 (±26.3)	53.1 (±25.4)	−1.0 (15.4)	0.217
SBP at rest (mmHg)	103 (±14)	110 (±14)	+7 (±17.4)	<0.0001
DBP at rest (mmHg)	55 (±11)	61 (±11)	+5.2 (±13)	<0.0001
SBP at 3 min exercise (mmHg)	124 (±18)	129 (±18)	4.8 (±11)	<0.0001
SBP at peak (mmHg)	154 (±23)	166 (±21)	11.7 (±24)	<0.0001
HR at rest (bpm)	68 (±11)	69 (±13)	0.4 (±11)	<0.0001
Peak exercise HR (bpm)	190 (±8)	187 (±8)	−3.6 (±7.4)	<0.0001
% predicted maximum HR for age	92.3 (±4)	91.7 (±3.9)		n.s.
Maximal workload (METs)	18.6 (±2.9)	18.7 (±3.1)		n.s.

Change in values and between visits are presented in mean± SD or (%). SBP is systolic blood pressure; DBP is diastolic blood pressure; HR is heart rate; BMI indicates body mass index; bpm, beats per minute; %, maximum predicted HR for age; * BMI percentiles: excluding subjects who had turned 18 years of age during follow-up, for whom percentiles were not calculated.

**Table 2:** Partial correlation analysis for between-visit variation of systolic blood pressure recorded at 3rd minute of exercise (ΔSBP_3min_).

	r	*p*
Male sex	0.30	<0.01
Number of practiced sports	0.11	<0.05
BMI at baseline (visit 1)	0.24	<0.01
BMI variation between-visit	0.18	<0.01
Classification of hypertension		n.s.

Data adjusted for SBP_3min_ at baseline, age at baseline, and follow-up duration.

**Table 3:** Partial correlation analysis for between-visit variation of systolic blood pressure recorded at exercise peak (ΔSBP_peak_).

	R	*p*
Male sex	0.38	<0.01
Baseline resting HR	−0.19	<0.01
BMI at baseline (visit 1)	0.13	<0.05
BMI variation between-visit	0.21	<0.01
Classification of hypertension		n.s.

Data adjusted for SBP_peak_ at baseline, age at baseline, and follow-up duration.

**Table 4:** Logistic regression analysis for independent determinants of higher quartiles of between-visit variation in systolic blood pressure recorded at 3rd minute of exercise (ΔSBP_3min)_ and in systolic blood pressure recorded at exercise peak (ΔSBP_peak_) Data adjusted for, age, follow-up duration, and BMI at baseline.

**ΔSBP_3min_**		
	ΔBMI	*p* < 0.001
**ΔSBP_peak_**		
	Male sex	*p* < 0.001
	ΔBMI	*p* < 0.001

## Data Availability

The raw data supporting the conclusions of this article will be made available by the authors on request.

## Abbreviations

The following abbreviations are used in this manuscript:

SBP_rest_	Systolic blood pressure at rest
SBP_3min_	Systolic blood pressure at 3rd minute of exercise
SBP_peak_	Systolic blood pressure at exercise peak
BMI	Body mass index
ESC	European Society of Cardiology
BP	Blood pressure
CV	Cardiovascular
RPE	Rate of perceived exertion
METs	Metabolic equivalents
EACPR	European Association of Cardiovascular Prevention and Rehabilitation
ROC	Receiver operating characteristic
